# Selective inhibition of host cell signaling for rotavirus antivirals: PI3K/Akt/mTOR-mediated rotavirus pathogenesis

**DOI:** 10.1080/21505594.2017.1356539

**Published:** 2017-08-18

**Authors:** Jason Kindrachuk

**Affiliations:** Laboratory of Emerging and Re-Emerging Viruses, Department of Medical Microbiology, University of Manitoba, Winnipeg, MB, Canada

**Keywords:** autophagy, cell signaling, drug repurposing, pathogenesis, rotavirus

In spite of over 40 years of basic and clinical research on rotaviruses following their discovery in 1973, rotaviruses continue to be a global health concern in children. Rotavirus, a member of the *Reoviridae* family of viruses, is the most common infectious agent underlying severe diarrheal disease in children^1^ and is responsible for approximately one-third of all cases requiring hospitalization.^2^ Rotavirus enteritis results in >500,000 deaths annually in children less than 5 y according to World Health Organization estimates.^3^ Although 2 licensed oral, live, attenuated rotavirus vaccines are available internationally (Rotarix and RotaTeq) >90% of rotavirus-mediated childhood fatalities occur within developing nations due to limited access to the vaccines, sanitation concerns and insufficient availability to routine healthcare services.^4^ Clinical management for acute rotavirus enteritis largely consists of oral rehydration therapy and zinc supplementation as there are no licensed antiviral therapies approved for rotavirus infections. As rotaviruses also cause severe diarrheal disease in young animals, including livestock species, they also represent a considerable threat to global livestock health with the potential for severe economic consequences. Increased clarity regarding the relation between rotavirus molecular pathogenesis and pathophysiology could help inform current patient care modalities. Further, the identification of key molecular determinants involved in the pathophysiology of severe rotavirus infections may also assist drug discovery and development strategies.

To this end there have been considerable efforts over the past 2 decades to identify the role of host-rotavirus interactions in rotavirus pathogenesis. In particular, many efforts have focused on how rotaviruses usurp host cell innate immune responses and the ubiquitous network of pattern recognition receptors (PRR). Rotaviruses are capable of suppressing interferon (IFN) responses during the early stages of infection.^5-8^ Indeed, prophylactic administration of IFN restricts rotavirus replication both *in vitro* and *in vivo*.^9-11^ Previous investigations have demonstrated that rotavirus infection does not result in PRR-mediated activation of IFNβ suggesting that viral-centric mechanisms exist to counteract these responses.^5,7,12-13^ The rotavirus nonstructural protein NSP1 mediates the degradation of multiple interferon regulatory factor (IRF) members including IRF3, IRF5 and IRF7.^5-6,8^ Further, it has also been suggested that NSP1 inhibits the nuclear translocation of the transcription factor signal transducer and activator of transcription 1 (STAT1).^14^ Recent investigations have also demonstrated that NSP1 may also downregulate innate immune responses through direct interactions with PRRs as NSP1 can downregulate retinoic acid-inducible gene 1 (RIG-I)-like receptors.^15^ In addition, there has been concerted efforts in the investigation of rotavirus-mediated regulation of host cell protein translation. It has been proposed that NSP3 modifies the intracellular localization of polyA-binding protein (PABP) by impairing its interaction with eIF4GI resulting in repression of host translation processes and accentuation of viral translation.^16-19^ Interestingly, recent work by Halasz et al. have demonstrated that rotavirus binding/entry and replication are increased via the induction of α2β1 and β2 integrin expression through a phosphatidylinositol 3-kinase (PI3K)-dependent mechanism.^20^

In this issue of Virulence, Yin et al. have provided compelling evidence for a role of the phosphatidylinositol 3-kinase (PI3K)/protein kinase B (Akt)/mechanistic target of rampamycin (mTOR) signaling pathway in productive rotavirus infection and have identified downstream signaling intermediates that may represent novel anti-rotavirus therapeutic targets.^21^ Here, the authors used *in vitro* cell culture models of rotavirus infection (simian rotavirus strain SA11) using traditional 2D cell culture (immortalized colorectal epithelial cells Caco2), 3D human primary intestinal organoids and pharmacologic inhibitors of the PI3K/Akt/mTOR pathway. Prophylactic treatment of immortalized cells and primary organoids with LY294002, a potent inhibitor of PI3K, inhibited total viral RNA and infectious virus particle production. Although these results suggest that PI3K could be an important target for future drug development considerations, the authors further assessed the roles of additional PI3K/Akt/mTOR signaling pathway intermediates in rotavirus infections. Inhibition of mTOR by shRNA or nanomolar concentrations of rapamycin (Sirolimus.Rapamune), a licensed mTOR inhibitor administered for the prevention of organ transplant rejection and lymphangioleiomyomatosis,^22^ resulted in significantly reduced rotavirus infection. These rapamycin-mediated inhibitory effects were retained following infection with 5 patient-derived rotavirus strains highlighting the broad importance of mTOR to productive rotavirus infection. Treatment with BEZ235, a dual PI3K/mTOR inhibitor, also inhibited rotavirus infection in both primary and immortalized cells. These observations build on previous investigations by Bagchi et al. regarding the importance of the PI3K/Akt/mTOR signaling pathway to rotavirus infection.^23-24^ Bagchi and colleagues demonstrated that rotavirus A5–13 infection results in activation of the PI3K/Akt signaling pathway through a nonstructural protein 1 (NSP1)-dependent mechanism. Yin et al. provide further confirmation of this phenomenon and provide novel information regarding downstream intermediates within the signaling pathway that are critical for productive rotavirus infections. Modulation of the PI3K/Akt/mTOR signaling pathway during productive infection has been demonstrated to be critical broad range of viruses that impact global health. A diverse range of viral families, including *Filoviridae* (Ebola virus),^25^ *Coronaviridae* (Middle East respiratory syndrome coronavirus),^26^ *Poxviridae* (monkeypox virus.cowpox virus.vaccinia virus),^27-28^ *Arenaviridae* (lymphocytic choriomeningitis virus) ^29^ and *Picornaviridae* (coxsackievirus) ^30^ require the activation of this pathway for productive infection.

Prior investigations have demonstrated that the induction of autophagy may represent an early defense mechanism within infected cells during viral infections. It is postulated that the induction of autophagy allows host cells to neutralize an invading pathogen early in the infectious cycle before the activation of apoptotic mechanisms within the infected cells.^31^ Multiple viruses, including human cytomegalovirus,^31^ hepatitis B virus-x ^32^ and chikungunya virus,^32^ have been shown to induce autophagy in infected cells. Recently, Wu et al. have demonstrated that rotavirus infection resulted in autophagy induction in the intestines of gnotobiotic pigs.^33^ Yin and colleagues have provided accompanying mechanistic data for rotavirus-induced autophagy. The authors demonstrated that silencing of eukaryotic translation initiation factor 4E-binding protein 1 (4E-BP1) resulted in a significant reduction in rotavirus infection as demonstrated by 4E-BP1 knockdown (immortalized cells) or deletion (4E-BP1 knockout mouse embryonic fibroblasts.MEF). Rapamycin treatment had no effect on rotavirus infection under these conditions. In contrast, rapamycin treatment inhibited rotavirus infection following reconstitution of 4E-BP1 in these cells. Building on these observations, Yin et al. demonstrated that knockdown of 1A/1B-light chain LC3-II, a microtubule-associated autophagosomal marker, and Beclin-1, which is involved in the early induction of autophagy.

Yin and colleagues have provided clear evidence for a central role of the PI3K/Akt/mTOR signaling pathway in rotavirus pathogenesis. Further, modulation of this pathway through selective inhibition of pathway intermediates results in inhibition of viral replication through a 4EB-P1-dependent induction of autophagy. Intriguingly, the authors' investigation suggests that licensed kinase inhibitors targeting the PI3K/Akt/mTOR pathway could potentially be repurposed as an alternative therapeutic strategy for combating rotavirus enteritis. The design and development of novel therapeutics, including anti-infectives, is increasingly impaired by the associated time and costs of development for moving novel therapeutics from pre-clinical phases to market. Repurposing of licensed therapeutics for alternative malignancies, including infectious diseases, is an enticing alternative to these impediments with recent support of this strategy from the National Institutes of Health Center for Advancing Translational Sciences.^34^ Thus, repurposing of licensed kinase inhibitors as novel anti-infective agents appears a logical approach. As of November, 2016, 31 kinase inhibitors have obtained US. Food and Drug Administration (FDA) licensure and there are continually increasing numbers in various clinical and pre-clinical stages of development.^35^ In support of this, the administration of miltefosine (an Akt inhibitor) has been granted approval for use as an antileishmanial agent.^36^ Our own investigations have demonstrated that pharmacologic inhibition of the PI3K/Akt/mTOR signaling pathway resulted in increased survival in a lethal model of Ebola virus infection in mice ^25^ and that inhibition of mTOR by rapamycin or everolimus inhibited Middle East respiratory syndrome coronavirus infection *in vitro*.^26^ Yin et al. provide compelling evidence for future *in vivo* efficacy studies of kinase inhibitors in rotavirus infection models. From a broader perspective, these results provide further justification for detailed investigations of the efficacy of selective inhibitors targeting the PI3K/Akt/mTOR signaling pathway as novel antiviral therapeutics for viruses that impact global human and/or livestock health.
